# E3 ligase ZFP91 inhibits Hepatocellular Carcinoma Metabolism Reprogramming by regulating PKM splicing

**DOI:** 10.7150/thno.44873

**Published:** 2020-07-09

**Authors:** De Chen, Yanjie Wang, Ruixun Lu, Xiaofeng Jiang, Xinhui Chen, Nan Meng, Min Chen, Shan Xie, Guang-Rong Yan

**Affiliations:** 1 Biomedicine Research Center, the Third Affiliated Hospital of Guangzhou Medical University, Guangzhou, 510150, China.; 2 Department of Surgery, the Second Affiliated Hospital of Guangzhou Medical University, Guangzhou, 510260, China.; 3 Key Laboratory of Protein Modification and Degradation, Guangzhou Medical University, Guangzhou, 511436, China.

**Keywords:** E3 ligase, Ubiquitination, Metabolism reprogramming, Hepatocellular carcinoma, ZFP91

## Abstract

**Rationale:** Hepatocellular carcinoma (HCC) is one of the most lethal cancers, and few molecularly targeted anticancer therapies have been developed to treat it. Thus, the identification of new therapeutic targets is urgent. Metabolic reprogramming is an important hallmark of cancer. However, how ubiquitin ligases are involved in the regulation of cancer metabolism remains poorly understood.

**Methods:** RT-PCR, western blot and IHC were used to determine ZFP91 expression. RNAi, cell proliferation, colony formation and transwell assays were used to determine the *in vitro* functions of ZFP91. Mouse xenograft models were used to study the *in vivo* effects of ZFP91. Co-IP together with mass spectrometry or western blot was utilized to investigate protein-protein interaction. Ubiquitination was analyzed using IP together with western blot. RNA splicing was assessed by using RT-PCR followed by restriction digestion. Lactate production and glucose uptake assays were used to analyze cancer metabolism.

**Results:** We identified that an E3 ligase zinc finger protein 91 (ZFP91) suppressed HCC metabolic reprogramming, cell proliferation and metastasis *in vitro* and *in vivo*. Mechanistically, *ZFP91* promoted the Lys48-linked ubiquitination of the oncoprotein hnRNP A1 at lysine 8 and proteasomal degradation, thereby inhibiting hnRNP A1-dependent PKM splicing, subsequently resulting in higher PKM1 isoform formation and lower PKM2 isoform formation and suppressing HCC glucose metabolism reprogramming, cell proliferation and metastasis. Moreover, HCC patients with lower levels of ZFP91 have poorer prognoses, and ZFP91 is an independent prognostic factor for patients with HCC.

**Conclusions:** Our study identifies ZFP91 as a tumor suppressor of hepatocarcinogenesis and HCC metabolism reprogramming and proposes it as a novel prognostic biomarker and therapeutic target of HCC.

## Introduction

HCC is one of the most common malignancies, exhibiting high metastasis and recurrence. Approximately 78.25 million new HCC cases and 74.55 million deaths occur worldwide every year, half of which occur in China 1. The prognosis of HCC patients remains poor, and the tumor recurrence five years after resection is more than 70% 2-4. Although current studies have found that advanced HCC patients who received the molecularly targeted drug sorafenib had a longer median survival than those taking the placebo 5, there are still many limitations in the targeted therapy of HCC at present. Therefore, it is imperative to explore the underlying mechanism responsible for HCC tumorigenesis to identify promising targets for HCC therapy.

Accumulating evidence has shown that cancer is an energy metabolism-related disease 6, 7. Metabolic reprogramming is an important hallmark of cancer. Cancer cells preferentially metabolize glucose to lactate despite the presence of oxygen 8, 9. This phenomenon is known as the Warburg effect or aerobic glycolysis, which provides selective advantages to cancer cells for cell proliferation, survival, migration and invasion by producing the increased metabolic intermediates essential for biosynthesis 10-12. Isoform selection of the glycolytic enzyme pyruvate kinase M (PKM) was demonstrated to switch the metabolism reprogramming of cancer cells 13, 14. PKM has two isoforms that were generated from alternative splicing of PKM pre-mRNA by the inclusion of exon 9 (PKM1) or exon 10 (PKM2). PKM1 is expressed in adult tissues to promote oxidative phosphorylation, while PKM2 is ubiquitously expressed in embryonic and tumor tissues and promotes aerobic glycolysis of tumor cells 15, 16. The related splicing regulators HOXB-AS3 peptide, hnRNP A1, hnRNP A2 and PTB notably contribute to PKM splicing 11, 14, 17-19.

The ubiquitin-proteasome system (UPS), which is responsible for degrading 80-90% of known proteins in cells, plays a vital role in cancer tumorigenesis and progression 20-24. The specificity of human UPS is mainly determined by approximately 617 E3 ubiquitin ligases. Some proteins (such as p53, p27 and cyclins) that are involved in cell cycle regulation and tumorigenesis are specifically regulated by E3 enzymes 25, 26. UPS components, including three enzyme families (E1, E2, and E3) and proteasomes, are potential therapeutic targets for cancer and other diseases 20, 27, 28.

ZFP91, a member of the zinc finger protein family that contains five zinc finger domains, was suggested to be a novel E3 ubiquitin ligase. ZFP91 was demonstrated to promote the Lys63-linked ubiquitination of NF-κB-inducing kinase (NIK) and stabilize NIK to activate the NF-κB pathway 29. ZFP91 was shown to be upregulated in human acute myelogenous leukemia (AML), prostate cancer and colon cancer 30-32. Further studies showed that ZFP91 promoted prostate cancer by activating the NF-κB pathway and colon cancer tumorigenesis and cell proliferation by activating the NF-κB-HIF1 pathway 31, 32. These findings suggest that ZFP91 is a potential oncogene. However, the functional roles of ZFP91 in cancer metabolism reprogramming have not been determined to date.

In this study, we found that ZFP91 was frequently downregulated in HCC compared to the corresponding adjacent nontumor liver tissues. The downregulation of ZFP91 was associated with HCC clinicopathological features and poor prognosis of patients with HCC. Knockdown of ZFP91 stimulated HCC cell proliferation, colony formation, migration, invasion, glucose uptake and lactate production *in vitro*, tumorigenesis and metastasis *in vivo*, while ZFP91 overexpression inhibited HCC progression and aerobic glycolysis. ZFP91 interacted with hnRNP A1 and Lys48-ubiquitinated hnRNP A1 at Lys 8 to promote proteasomal degradation of hnRNP A1, thereby inhibiting hnRNP A1-dependent PKM mRNA pre-mRNA splicing to induce PKM1 isoform formation and inhibit PKM2 isoform formation and HCC metabolism reprogramming. Collectively, our findings reveal that ZFP91 is a tumor suppressor in HCC, regulates the ubiquitination and degradation of a novel substrate, hnRNP A1, and suppresses HCC metabolism programming, cell proliferation and metastasis. ZFP91 may be an independent prognostic biomarker, as well as a potential therapeutic target for HCC.

## Results

### ZFP91 was frequently downregulated in HCC

**Figure 1:**
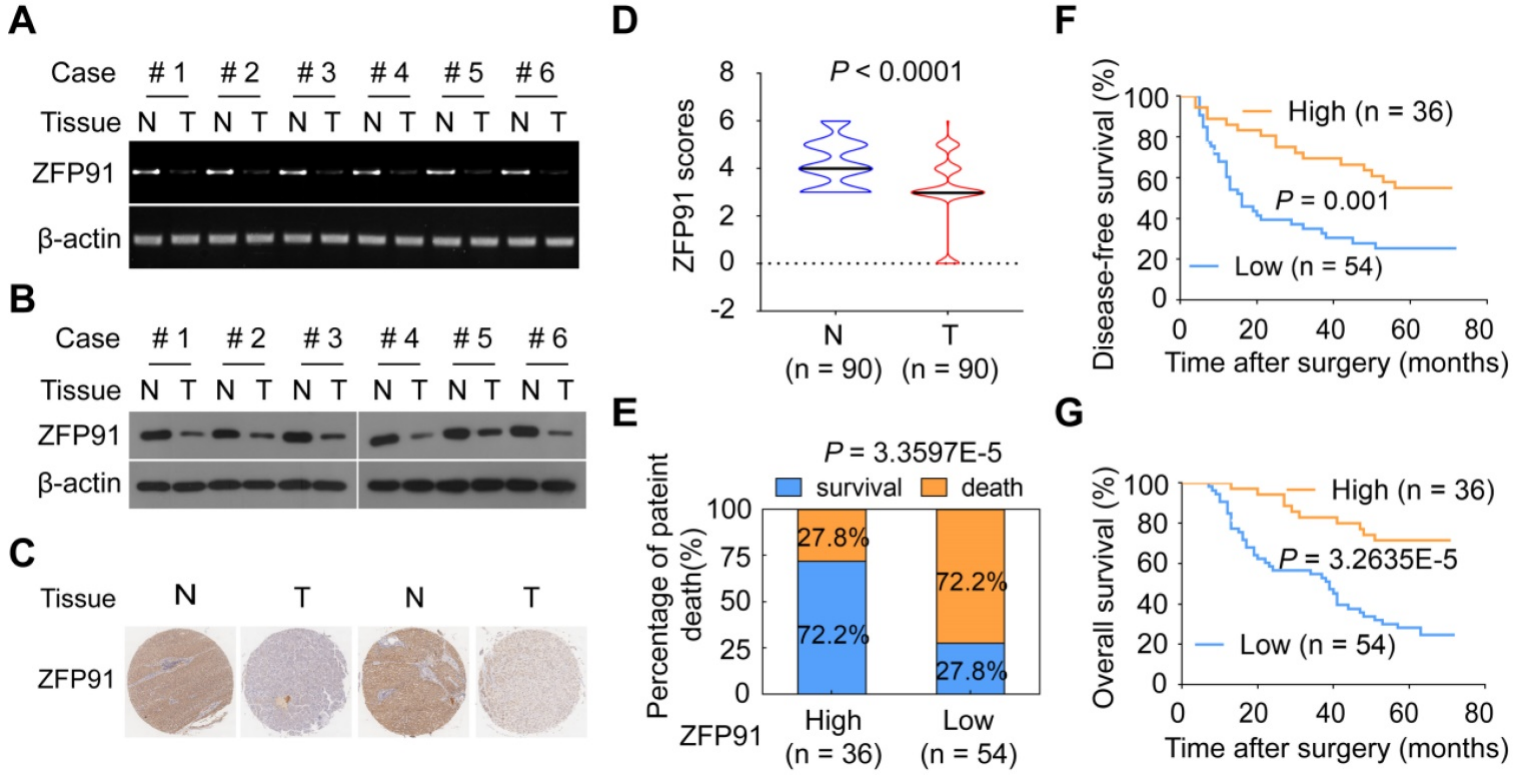
ZFP91 is frequently downregulated in HCC, and its downregulation is associated with a poor prognosis for patients with HCC. (**A, B**) The mRNA (A) and protein (B) levels of ZFP91 were detected in primary HCC tissues (T) and corresponding adjacent nontumoral liver (N) tissues. (**C**) Representative IHC images of ZFP91 protein expression in HCC tissues and their corresponding nontumoral liver tissues. (**D**) Differences in the ZFP91 protein level between HCC tissues (T) and their corresponding nontumoral liver tissues (N) are presented as a violin plot (n = 90). (**E**) Association between ZFP91 protein levels and the percentage of patient death in HCC samples. (**F, G**) The disease-free survival rate (F) and overall patient survival rate (G) for patients with HCC according to ZFP91 expression ratios of HCC/corresponding nontumoral liver tissues.

To investigate the role of *ZFP91* in HCC, we analyzed *ZFP91* mRNA and protein levels in six pairs of fresh primary HCC tissue samples and matched adjacent nontumoral hepatic tissue (N) samples. Interestingly, *ZFP91* mRNA and protein levels are downregulated in all six primary HCC tissue samples compared with matched adjacent nontumoral hepatic tissue samples (Figure 1A and 1B), which is inconsistent with previous studies in which ZFP91 expression is upregulated in AML, prostate and colon cancer tissues 30-32. Furthermore, an extensive tissue microarray analysis of 90 pairs of matched HCC and corresponding nontumoral liver tissue samples was performed using an IHC assay (Figure 1C). ZFP91 protein level was down-regulated in 59% of HCC tissue samples, unchanged in 25% of HCC tissue samples, up-regulated in 16% of HCC tissue samples, compared to their corresponding nontumoral liver tissue samples (*P* < 0.0001) (Figure 1D). Collectively, these data indicate that ZFP91 is frequently downregulated in HCC.

### Reduced ZFP91 levels were correlated with clinicopathological features and poor prognosis for patients with HCC

Furthermore, the correlations between the ZFP91 protein levels and the clinicopathological features of patients with HCC were analyzed in 90 HCC samples. The decrease of ZFP91 was positively associated with clinical stage (*P* = 0.010), TNM stage (*P* = 0.018) and recurrence of cancer (*P* = 0.023) in HCC (Table S1). The patients with HCC and low ZFP91 levels had a higher risk of cancer recurrence and death compared with those classified as high ZFP91 levels (Figure 1E). Kaplan-Meier survival analyses revealed that ZFP91 expression correlated significantly with disease-free survival rate (*P* = 0.001, log-rank test) (Figure 1F) and overall patient survival rate (*P* = 3.2635E-5, log-rank test) (Figure 1G). The mean overall survival time for patients with HCC and high ZFP91 expression (protein score ≥ 4) was 58 months, whereas that for patients with HCC and low ZFP91 (protein score < 4) was 39 months. Further multivariate Cox regression analysis showed that low ZFP91 expression is an independent prognostic factor for poor survival of HCC patients (HR = 0.32, 95% CI = 0.149-0.689, *P* = 0.004) (Table 1). Collectively, our findings indicate that a low ZFP91 level is significantly correlated with a poor prognosis for patients with HCC and that ZFP91 serves as an independent prognostic factor for patients with HCC. These data suggest that ZFP91 is a potential tumor suppressor in HCC.

### ZFP91 suppresses HCC cell proliferation and metastasis

**Figure 2:**
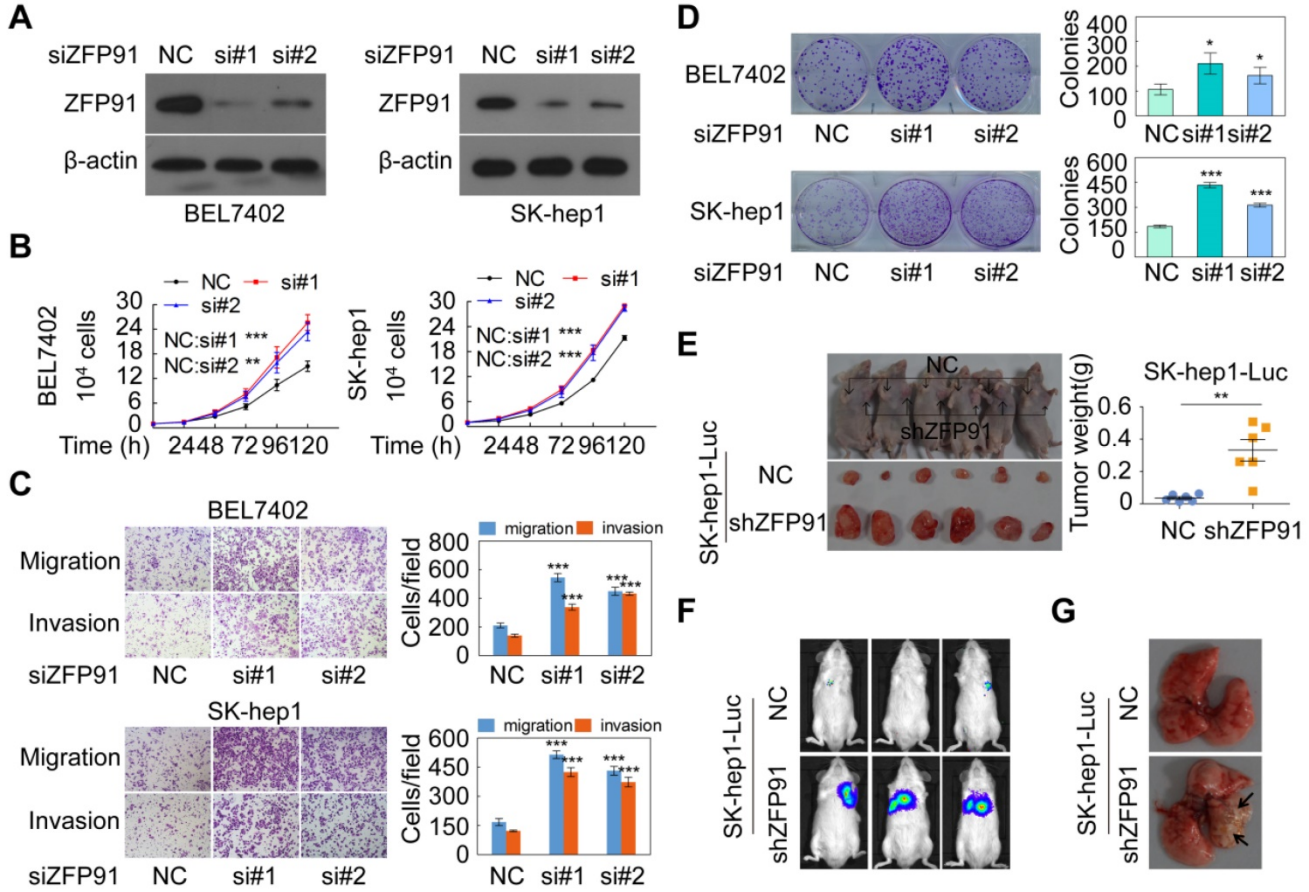
ZFP91 silencing promotes HCC cell growth, colony formation, migration and invasion *in vitro* and tumorigenesis and metastasis *in vivo*. (**A-D**) BEL7402 and SK-hep1 cells were transfected with anti-ZFP91 siRNAs; the indicated proteins (A), cell growth (B), migration and invasion (C), and colony formation (D) were determined. (**E**) The *in vivo* growth of the indicated cell lines stably silencing ZFP91 was examined. Mouse xenograft tumors are shown in the left panel. The weights of the xenograft tumors are presented in the right panel (n = 6). (**F, G**) Luc-labeled SK-hep1 cells (2 × 10^6^ cells/mouse) were injected into NOD-SCID mice; the luciferase activity was visualized 2 months posttransplantation (n = 5) (F), and the metastatic tumor nodules in mouse lung were observed (G). Data are represented as mean ± SD.

To investigate the functions of ZFP91 in HCC cell proliferation and metastasis, ZFP91 expression levels were determined in five HCC cell lines and three normal liver tissues. We found that ZFP91 expression was lower in all five HCC cell lines than in all three normal liver tissues, consistent with results in Figure 1 (Figure S1A). We selected HCC cell lines SK-hep1 and BEL7402 with relative high ZFP91 level to knockdown ZFP91 expression and selected HCC cell lines MHCC-LM3 and MHCC97H with relative low ZFP91 level to overexpress ZFP91. Silencing of ZFP91 stimulated cell growth, migration and invasion, and colony formation in HCC cells SK-hep1 and BEL7402 (Figure 2A-2D). In contrast, ZFP91 overexpression inhibited cell growth, migration and invasion, and colony formation in HCC cells MHCC-LM3 and MHCC97H (Figure S1B-S1E).

More importantly, the* in vivo* growth of HCC xenografts composed of SK-hep1 cells stably silenced with *ZFP91* was significantly increased compared with xenografts composed of negative control SK-hep1 cells (Figure 2E). In addition, the mice injected with luciferase-tagged SK-hep1 cells with stable silencing of *ZFP91* by tail vein showed larger metastatic nodules and tumorigenesis in their lungs compared with those composed of luciferase-tagged control SK-hep1 cells (Figure 2F and 2G). Taken together, our results demonstrate that *ZFP91* inhibits HCC cell proliferation and metastasis.

### ZFP91 promotes the K48 ubiquitination and degradation of hnRNP A1

**Figure 3:**
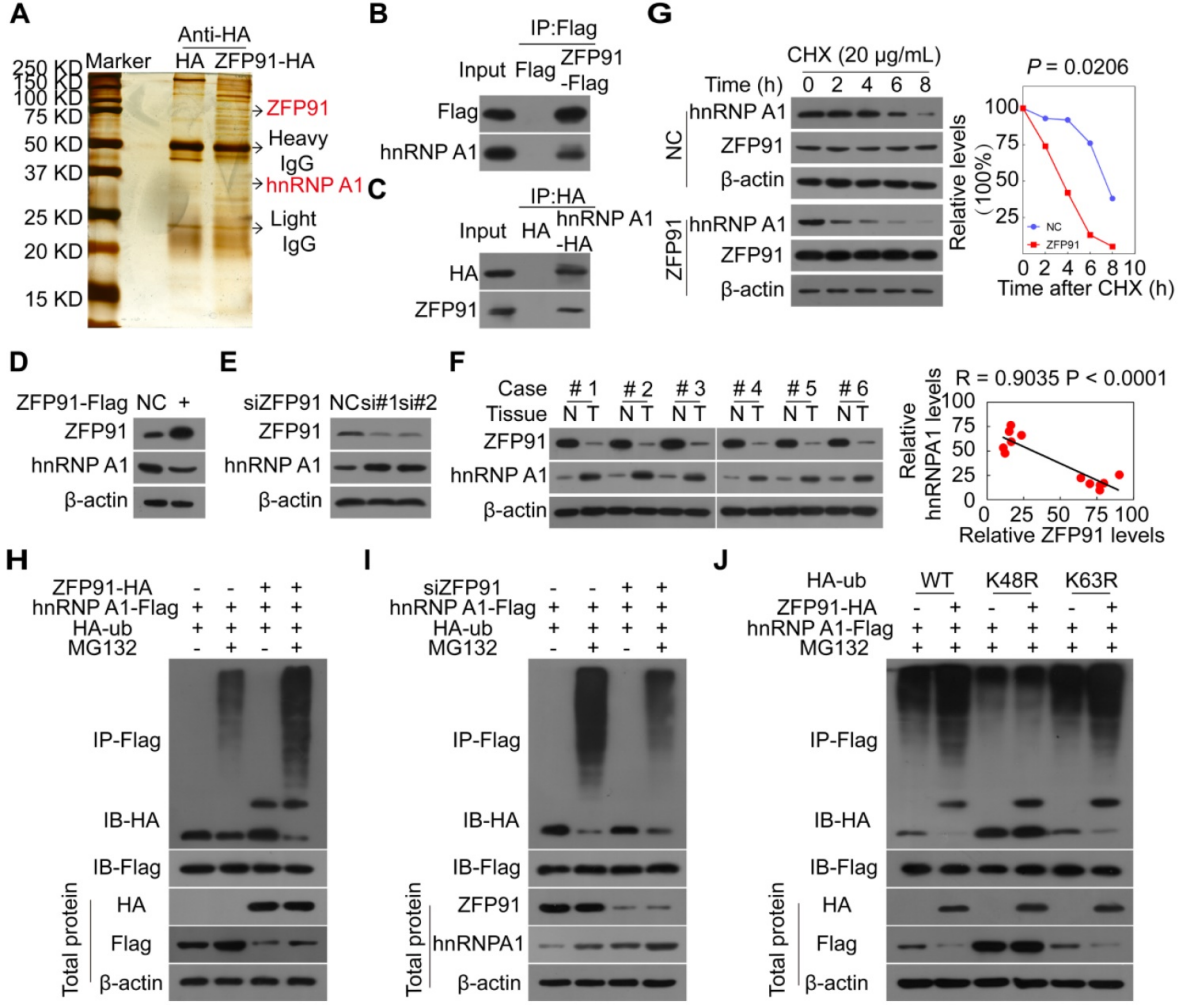
ZFP91 K48-ubiquitinates and degrades hnRNP A1. (**A**) Proteins that interacted with ZFP91 were identified by co-IP and mass spectrometry assays using HEK293T cells as a cell model. (**B, C**) ZFP91-Flag and hnRNP A1-HA plasmids were transfected into HCC cells HCC MHCC-LM3 (B) and SK-hep1 (C), ZFP91-Flag (B) and hnRNP A1-HA (C) complexes were co-immunoprecipitated with anti-Flag and HA antibodies, and hnRNP A1 and ZFP91 were detected, respectively. (**D**) MHCC-LM3 HCC cells were transfected with ZFP91-Flag plasmid, hnRNP A1 protein level was determined. (**E**) SK-hep1 HCC cells were transfected with anti-ZFP91 siRNA, hnRNP A1 protein level was determined. (**F**) ZFP91 and hnRNP A1 protein levels in six pairs of primary HCC tissues (T) and corresponding N tissues used in Figure 1B were detected. The correlations of ZFP91 with hnRNP A1 were analyzed in the right panel. (**G**) MHCC-LM3 cells transfected with ZFP91-Flag plasmids for 36 h were incubated with cycloheximide (CHX) for the indicated times. The indicated proteins were analyzed (left panel), and the relative hnRNP A1 protein level is illustrated graphically (right panel). (**H**) MHCC-LM3 cells were cotransfected with the indicated plasmids for 36 h, followed by treatment with 10 µM MG132; the polyubiquitination level of hnRNP A1 was detected. (**I**) SK-hep1 cells were cotransfected with the indicated plasmids together with anti-ZFP91 siRNA for 36 h, followed by treatment with MG132; the polyubiquitination level of hnRNP A1 was detected. (**J**) MHCC-LM3 cells were cotransfected with wild type HA-ub or its mutants together with ZFP91-HA and hnRNP A1-Flag plasmids for 36 h, followed by treatment with MG132; the polyubiquitination level of hnRNP A1 was detected.

To investigate the molecular mechanism by which ZFP91 suppresses HCC cell proliferation and metastasis, we identified the proteins that interacted with ZFP91 to find the substrates regulated by E3 ligase ZFP91, by performing Co-IP and mass spectrometry analyses (Figure 3A). Previous studies have found that ZFP91 is upregulated in AML, prostate and colon cancer tissue and regulates the K63 ubiquitination of NIK 31, 32. In this study, we did not identify the NIK protein. Among the identified ZFP91-interacted proteins, hnRNP A1 is particularly interesting because hnRNP A1 is an important oncoprotein and a critical determinant of the glycolytic enzyme pyruvate kinase M (PKM) splicing 11, 14, 17, 33, 34. We further confirmed that hnRNP A1 was present in ZFP91 complexes (Figure 3B) and ZFP91 was confirmed to exist in hnRNP A1 complexes (Figure 3C), indicating that hnRNP A1 may be a potential substrate of E3 ligase ZFP91.

To determine whether ZFP91 is responsible for hnRNP A1 ubiquitination, we investigated the effects of ZFP91 on hnRNP A1 mRNA, protein levels, half-life and ubiquitination. We found that *ZFP91* overexpression decreased hnRNP A1 protein levels but not *hnRNP A1* mRNA levels, while silencing of *ZFP91* increased hnRNP A1 protein levels but not *hnRNP A1* mRNA levels (Figure 3D and 3E, Figure S2A and S2B). The hnRNP A1 protein levels were increased in HCC tissues compared to in the matched adjacent nontumoral liver tissues, whereas ZFP91 levels were downregulated in HCC (Figure 3F). And the hnRNP A1 protein levels were also increased in mouse xenograft tumors composed of ZFP91-silenced HCC cells compared to in mouse xenograft tumors composed of ZFP91-unsilenced HCC cells (Figure S2C). A relative high hnRNP A1 level was also observed in HCC cell line with the relative low ZFP91 expression (Figure S1A). Consistently, these results strongly indicated that hnRNP A1 protein level was negatively correlated with ZFP91 level (Figure 3F and Figure S2C).

Furthermore, we found that *ZFP91* overexpression resulted in a shorter hnRNP A1 protein half-life (Figure 3G). The *in vivo* ubiquitination assay revealed that *ZFP91* overexpression increased hnRNP A1 polyubiquitination levels in the presence of the specific proteasome inhibitor MG132 (Figure 3H), while ZFP91 knockdown decreased the polyubiquitination level of hnRNP A1 in the presence of MG132 (Figure 3I). Our previous study showed that ZFP91 is a downstream effector of ECD and ECD blocks the polyubiquitination and degradation of hnRNP F of ZFP91 in gastric cancer 35. We here found that ECD blocked the interaction of ZFP91 with hnRNP A1 and the polyubiquitination of ZFP91 on hnRNP A1 (Figure S2D and S2E).

To investigate which ubiquitin linkage species (K48 versus K63) on hnRNP A1 were catalyzed by ZFP91, we mutated the lysine 48 (K48) and 63 (K63) at ubiquitin to arginine, two ubiquitin mutants K48R and K63R were constructed. ZFP91 did not promote the polyubiquitination of hnRNP A1 when the ubiquitin at K48 was mutated, while ZFP91 still promoted the polyubiquitination of hnRNP A1 when the ubiquitin at K63 was mutated (Figure 3J). In summary, our results indicate that ZFP91 promotes the K48 ubiquitination and degradation of the hnRNP A1 protein.

### ZFP91 ubiquitinates hnRNP A1 at Lys8

**Figure 4:**
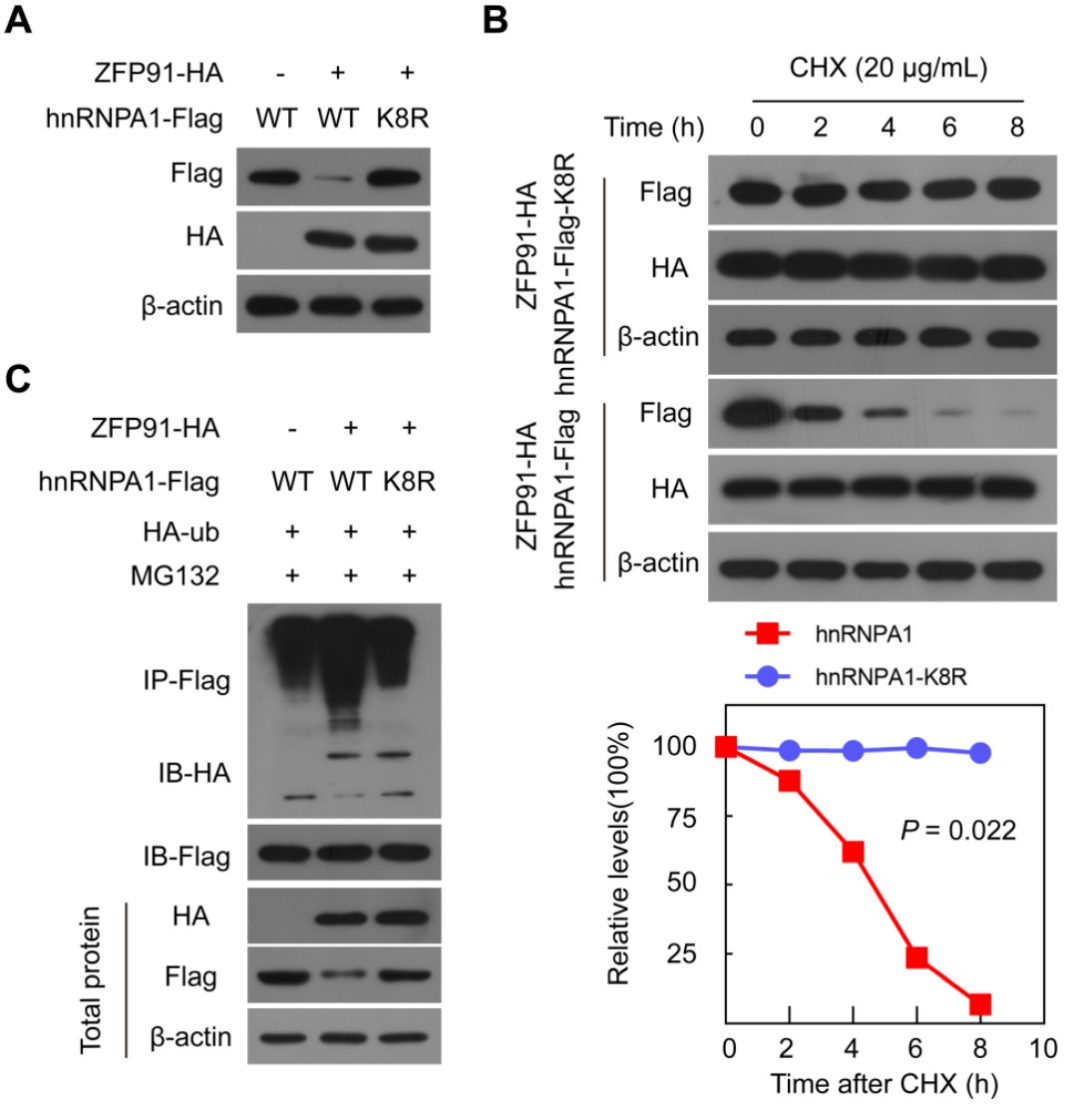
ZFP91 ubiquitinates hnRNP A1 protein at Lys8. (**A**) The ZFP91-HA plasmids together with wild type hnRNP A1-Flag plasmids or hnRNP A1-Flag K8R mutant were cotransfected into MHCC-LM3 cells for 36 h, and the hnRNP A1-Flag protein level was determined. (**B**) MHCC-LM3 cells were cotransfected with the ZFP91 plasmids together with wild type hnRNP A1-Flag plasmids or hnRNP A1-Flag K8R mutant for 36 h and then incubated with CHX for the indicated times. The indicated proteins were determined (top panel), and the relative hnRNP A1-Flag protein level is illustrated graphically (bottom panel). (**C**) MHCC-LM3 cells were cotransfected with the indicated plasmids for 36 h and then treated with 10 µM MG132. The polyubiquitination levels of hnRNP A1-Flag and hnRNP A1-Flag K8R mutant protein were analyzed.

We further investigated and identified the ubiquitination site on the hnRNP A1 protein. HnRNP A1-Flag protein was immunoprecipitated using an anti-Flag antibody, and the ubiquitination modification of lysine residues on hnRNP A1 was identified by mass spectrometry and found that Lys 8 on hnRNP A1 was ubiquitination-modified (Figure S3). To validate the specific ubiquitination modification of Lys 8 sites on the hnRNP A1 protein, we constructed the *hnRNP A1* mutant, in which Lys8 was substituted with arginine (hnRNP A1 K8R). When *ZFP91* was coexpressed with wild-type *hnRNP A1* or *hnRNP A1 K8R* in cells, ZFP91 decreased the wild-type hnRNP A1 protein level but had no obvious effect on the hnRNP A1 K8R protein level (Figure 4A). Furthermore, *ZFP91* overexpression did not result in a shorter hnRNP A1 K8R protein half-life compared to wild type hnRNP A1 (Figure 4B). The *in vivo* ubiquitination assay also showed that ZFP91 promoted the polyubiquitination level of wild-type hnRNP A1 but had no effect on the polyubiquitination level of hnRNP A1 K8R (Figure 4C). Collectively, these results indicate that ZFP91 promotes the degradation of hnRNP A1 by ubiquitinating hnRNP A1 at Lys8.

### Overexpression of ZFP91 impairs hnRNP A1 functions in HCC cells

As expected, overexpression of *hnRNP A1* promoted HCC cell growth, migration and invasion and colony formation (Figure S4A-S4D), while silencing of *hnRNP A1* suppressed HCC cell malignant phenotypes (Figure S4E-S4H), indicating that hnRNP A1 is a driver for HCC cell proliferation and metastasis, which is contrary to the functions of ZFP91.

**Figure 5:**
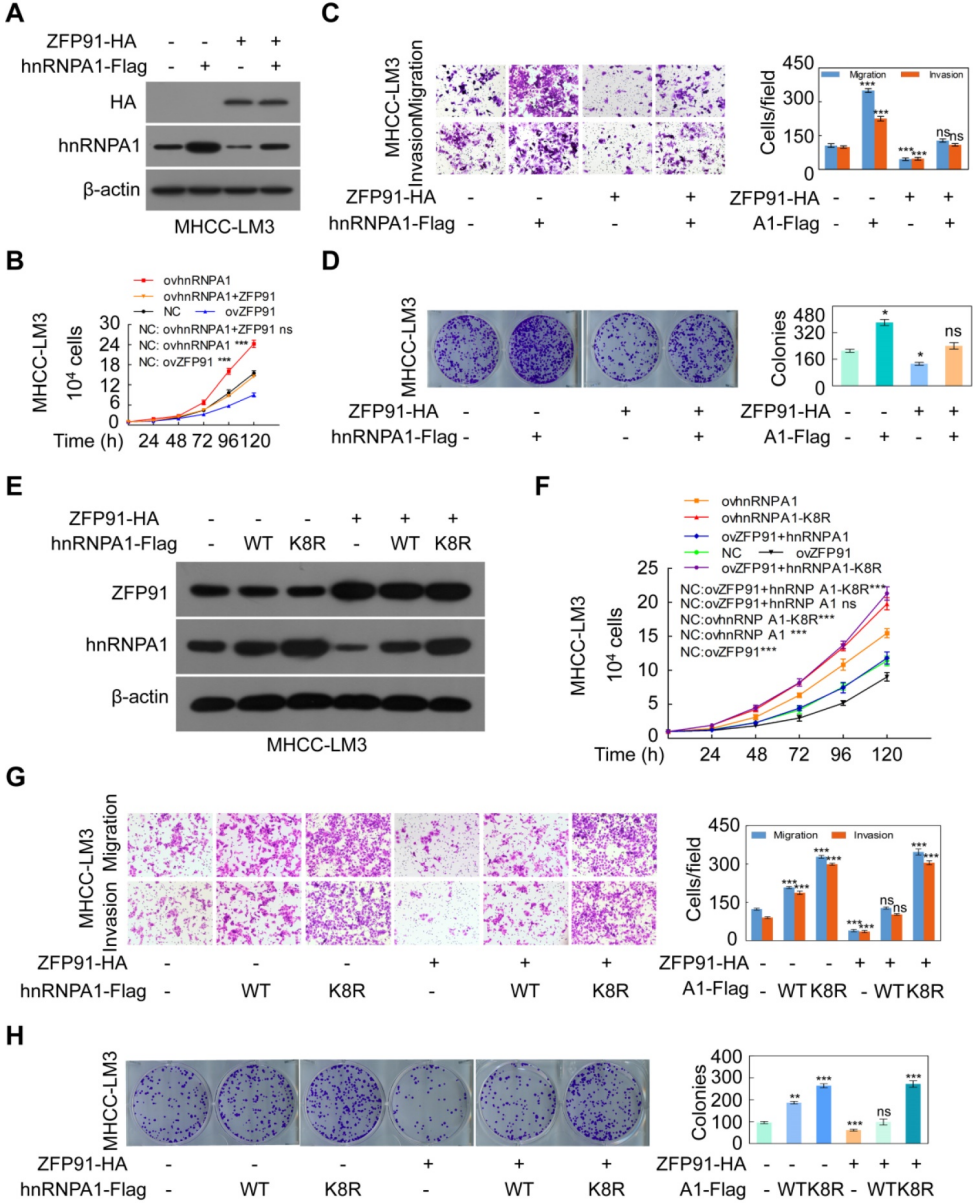
ZFP91 inhibits HCC cell proliferation, colony formation, migration and invasion by ubiquitinating hnRNP A1 at Lys8 to induce the ubiquitin-mediated degradation of hnRNP A1. (**A-D**) Both ZFP91-HA and hnRNP A1-Flag plasmids were cotransfected into HCC MHCC-LM3 cells; the indicated proteins (A), migration and invasion (B), cell growth (C) and colony formation (D) were detected (n = 3). (**E-H**) HCC MHCC-LM3 cells were cotransfected with the ZFP91-HA plasmids together with wild type hnRNP A1-Flag plasmids or hnRNP A1-Flag K8R mutant; the indicated proteins (E), migration and invasion (F), cell growth (G) and colony formation (H) were detected (n = 3). Data are represented as mean ± SD.

To confirm that ZFP91 suppresses HCC cell proliferation and metastasis through hnRNP A1, *hnRNP A1* together with *ZFP91* were cotransfected into HCC MHCC-LM3 cells. The ectopic expression of *ZFP91* antagonized the enhancement of HCC cell growth, colony formation, migration and invasion induced by *hnRNP A1* overexpression (Figure 5A-5D). Furthermore, while the wild-type *hnRNP A1* vector or *hnRNP A1 K8R* mutant together with *ZFP91* was cotransfected into HCC MHCC-LM3 cells, ZFP91 did not impair the HCC cell malignant phenotypes induced by hnRNP A1 K8R overexpression but not by wild-type hnRNP A1 overexpression because ZFP91 could ubiquitinate and degrade wild-type hnRNP A1 protein but not hnRNP A1 K8R mutant protein in which the ZFP91-regulated ubiquitination lysine site was mutated in hnRNP A1 protein (Figure 5E-5H). Taken together, these results indicate that ZFP91 inhibits HCC cell proliferation and metastasis by ubiquitinating and degrading the oncoprotein hnRNP A1 at Lys8.

### ZFP91 regulates *PKM* pre-mRNA splicing via hnRNPA1

**Figure 6:**
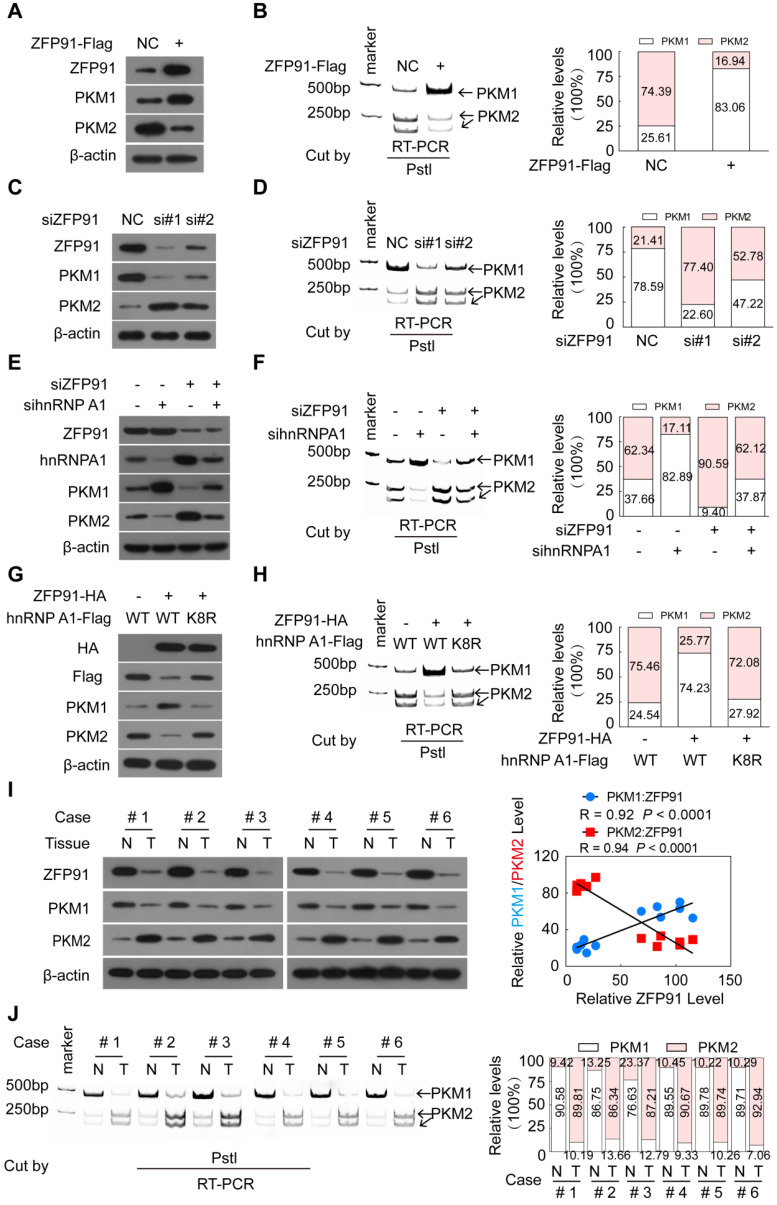
ZFP91 regulates the alternative splicing of *PKM* pre-mRNA to promote PKM1 isoform formation and inhibit PKM2 isoform formation by ubiquitinating at Lys 8 and degrading hnRNP A1. (**A-D**) HCC MHCC-LM3 and SK-hep1 cells were transfected with ZFP91-Flag plasmids (A, B) or anti-ZFP91 siRNAs (C, D), respectively; the protein levels of PKM1 and PKM2 (A, C) and the PKM pre-mRNA splicing (B, D) were detected. (**E, F**) HCC SK-hep1 cells were cotransfected with anti-ZFP91 siRNA together with anti-hnRNP A1 siRNA; the protein levels of PKM1 and PKM2 (E) and PKM pre-mRNA splicing (F) were detected. (**G, H**) MHCC-LM3 cells were cotransfected with the ZFP91-HA plasmids together with wild type hnRNP A1-Flag plasmids or hnRNP A1-Flag K8R mutant; the protein levels of PKM1 and PKM2 (G) and PKM pre-mRNA splicing (H) were detected. (**I, J**) The protein levels of PKM1 and PKM2 (I) and PKM pre-mRNA splicing (J) were detected in six pairs of primary HCC tissues (T) and corresponding adjacent nontumoral liver tissues (N) used in Figure 1B. The correlations of ZFP91 protein levels with PKM1 and PKM2 protein levels were analyzed in clinical HCC samples (right panel in I).

HnRNP A1 is a regulator responsible for alternative splicing of* PKM* pre-mRNA, and suppresses the inclusion of *PKM* exon 9 to induce PKM2 isoform formation and inhibits PKM1 isoform formation 11, 14, 17-19. Thus, we further investigated whether ZFP91 regulates alternative splicing of *PKM* pre-mRNA. We found that ZFP91 overexpression decreased the mRNA and protein levels of the *PKM2* isoform and increased the mRNA and protein levels of the *PKM1* isoform (Figure 6A and 6B). In contrast, silencing of ZFP91 resulted in the opposite phenomenon (Figure 6C and 6D).

As expected, hnRNP A1 overexpression promoted PKM2 isoform formation and inhibited PKM1 isoform formation (Figure S5A and S5B), while knockdown of hnRNP A1 resulted in the opposite effects (Figure S5C and S5D). HnRNP A1 knockdown blocked the enhancement of PKM2 isoform protein and mRNA level and the reduction of PKM1 isoform protein and mRNA level induced by ZFP91 knockdown (Figure 6E and 6F). However, the ectopic expression of ZFP91 antagonized the enhancement of PKM2 isoform formation and the decrease of PKM1 isoform formation induced by hnRNP A1 overexpression (Figure S5E and S5F). More importantly, the ectopic expression of *ZFP91* did not impair the enhancement of PKM2 isoform formation and the decrease of PKM1 isoform formation induced by *hnRNP A1 K8R* mutant overexpression but not by wild-type *hnRNP A1* overexpression (Figure 6G and 6H), indicating that ZFP91 regulates PKM splicing by ubiquitinating and degrading hnRNP A1.

The PKM1 and PKM2 levels and the relationship between ZFP91 levels and PKM1 and PKM2 levels in five pairs of mouse xenograft tumors in Figure 2E were further investigated. The mRNA and protein levels of the PKM1 and PKM2 isoforms were decreased and increased in all ZFP91-silenced mouse xenograft tumors compared to those in the negative control (Figure S5G and S5H). The PKM1 and PKM2 protein levels were positively (R = 0.92, *P* < 0.0001) and negatively (R = 0.9434, *P* < 0.0001) correlated with ZFP91 protein levels in mouse xenograft tumors, respectively (Figure S5G).

Finally, the PKM1 and PKM2 levels and the relationship between ZFP91 levels and PKM1 and PKM2 levels in clinical human HCC tissue samples were determined. The mRNA and protein levels of *PKM1* were decreased in all six HCC tissues compared to their corresponding adjacent nontumor liver tissues, while the mRNA and protein levels of *PKM2* were increased (Figure 6I and 6J). A similar change was also observed in five HCC cell lines and normal liver tissues (supplementary Figure S1A). PKM1 levels were positively correlated with ZFP91 levels (R = 0.926, *P* = 0.0001), while PKM2 levels were negatively correlated with ZFP91 protein levels (R = 0.9337,* P* **<** 0.0001) in HCC tissue samples (Figure 6I). Collectively, these results demonstrate that ZFP91 regulates *PKM* splicing to induce PKM1 isoform formation and inhibit PKM2 isoform formation by ubiquitinating and degrading hnRNP A1.

### ZFP91 inhibits the Warburg effect in HCC cells

**Figure 7:**
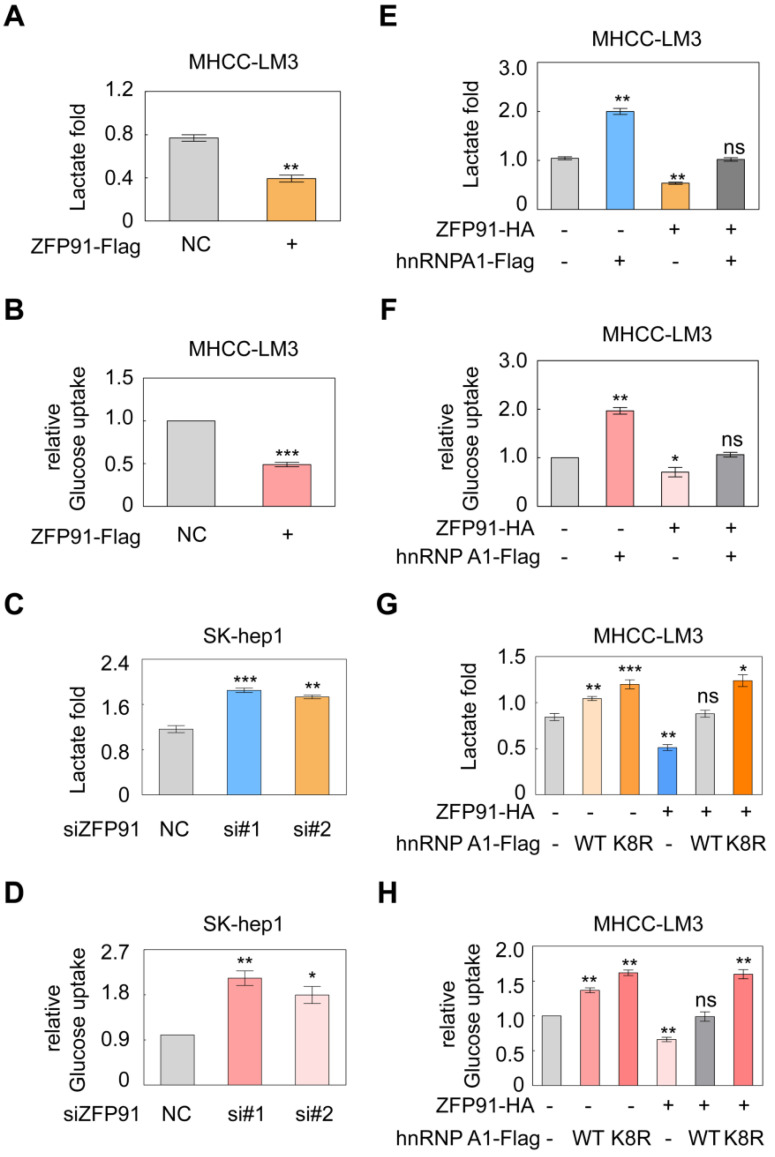
ZFP91 inhibits HCC cell aerobic glycolysis by ubiquitinating and degrading hnRNP A1. (**A, B**) MHCC-LM3 cells were transfected with ZFP91-Flag plasmids; lactate production (A) and glucose uptake (B) were measured (n = 3). (**C, D**) SK-hep1 cells were transfected with anti-ZFP91 siRNAs, lactate production (C) and glucose uptake (D) were measured (n = 3). (**E, F**) ZFP91-HA vectors and hnRNP A1-Flag plasmids were cotransfected into MHCC-LM3 cells; lactate production (E) and glucose uptake (F) were measured (n = 3). (**G, H**) MHCC-LM3 cells were cotransfected with the ZFP91-HA plasmids together with wild type hnRNP A1-Flag or hnRNP A1-Flag K8R vector, lactate production (G) and glucose uptake (H) were measured (n = 3). Data are represented as mean ± SD.

PKM2 isoform formation is a critical determinant of the Warburg effect in cancer cells 16, 36. The Warburg effect, which is characterized by increased glucose uptake and lactate production, provides a selective advantage for cancer tumorigenesis and progression. Therefore, we further investigated the influence of *ZFP91* on the Warburg effect in HCC cells. We revealed that *ZFP91* overexpression significantly decreased lactate production and glucose uptake in HCC cells (Figure 7A and 7B), whereas silencing of *ZFP91* increased lactate production and glucose uptake (Figure 7C and 7D).

To further investigate whether ZFP91 inhibits the Warburg effect by ubiquitinating and degrading hnRNP A1, we coexpressed *ZFP91* and *hnRNP A1* in HCC cells. We found that *ZFP91* overexpression antagonized the enhancement of lactate production and glucose uptake induced by *hnRNP A1* overexpression (Figure 7E and 7F). However, when *ZFP91* was coexpressed with wild-type *hnRNP A1* or *hnRNP A1 K8R* in HCC cells, *ZFP91* overexpression did not antagonize the increased Warburg effect induced by *hnRNP A1 K8R* mutant overexpression because ZFP91 did not ubiquitinate and degrade hnRNP A1 K8R mutant protein (Figure 7G and 7H). Taken together, these results show that ZFP91 suppresses HCC cell metabolism reprogramming by ubiquitinating and degrading hnRNP A1 protein.

### ZFP91 inhibits HCC progression through PKM2

To investigate whether ZFP91 inhibits HCC progression through PKM2, ZFP91 and PKM2 expressions were knocked-down in HCC cells. We found that the enhancement of HCC cell proliferation, colony formation, migration and invasion induced by ZFP91 depletion was completely impaired by PKM2 knockdown (Figure S6), indicating that ZFP91 inhibiting HCC progression through PKM2. Taken together, our results indicate that ZFP91-hnRNP A1-PKM2 axis suppresses HCC cell metabolism re-programming, cell proliferation and metastasis.

## Discussion

In this study, we found that ZFP91 is a suppressor for HCC aerobic glycolysis, cell proliferation and metastasis by K48-ubiquitinating and degrading oncoprotein hnRNP A1, expanding upon its previously reported role as an oncogene in AML, prostate and colon cancers in which ZFP91 promotes cancer cell proliferation and tumorigenesis by ubiquitinating and stabilizing NIK to activate the NF-κB pathway 29-32. We found that ZFP91 levels were downregulated in HCC, whereas ZFP91 expression was upregulated in AML, prostate and colon cancers 30-32. Whether ZFP91 also regulates the K48 ubiquitination and degradation of hnRNP A1 in AML, prostate and colon cancers and other cancers with ZFP91 expression upregulation remains to be explored in the future. Previous studies have shown that some genes are oncogenes in several cancers, whereas they are tumor suppressors in several cancers. For example, GSK-3β is classically recognized as a tumor suppressor in a variety of tumors, whereas GSK-3β is considered an oncogene and actually promotes the development of several other cancer types, including mixed lineage leukemia 37, 38, glioma 39, osteosarcoma 40, and oral cancer 41. Therefore, the biological function roles of ZFP91 require assessment in each type of tumor in the future.

In addition, we analyzed the ZFP91 expression in Human Protein atlas from TCGA database, and found that high ZFP91 mRNA level showed marginal unfavorable impact on survival for patients with HCC, inconsistent with our finding in which low ZFP91 protein shows unfavorable impact on survival for patients with HCC. The possible cause of the inconsistence is: In Human Protein atlas, ZFP91 RNA levels were analyzed in HCC patients of American including white (51.27%), Asian (43.91%), and black or African American (4.82%), while in this study, ZFP91 protein levels were analyzed in HCC patient of China. It is well known that the pathogenesis of liver cancer in Chinese and American are different. The pathogenesis of HCC in American is mainly induced by high fat diet, while the pathogenesis of HCC in Chinese is mainly induced by HBV and HCV infection.

In a role in promoting tumorigenesis, ZFP91 Lys 63-ubiquitinates and stabilizes NIK to activate the NF-κB pathway, thereby promoting cancer cell proliferation and tumorigenesis 29, 31, 32. In this study, a novel ubiquitin substrate, hnRNP A1, of the E3 ligase ZFP91 was discovered. Furthermore, we found that ZFP91 Lys 48-ubiquitinates hnRNP A1 at Lys8 and contributes to the ubiquitin-mediated proteolysis of hnRNP A1. Our findings show that ZFP91 not only promotes the stabilization of its substrates through ubiquitination modification but also results in the degradation of its substrates in a ubiquitination-dependent manner. Therefore, our findings provide a novel regulatory of ZFP91 in cancer and enrich ZFP91 roles and mechanisms in cancer.

In this study, we found that ZFP91 regulates the K48 ubiquitination of hnRNP A1 at Lys8. However, hnRNP A1 is still partially ubiquitination-modified when Lys 8 was mutated at hnRNP A1, indicating that the additional ubiquitnation sites, which is not regulated by ZFP91, is existed on hnRNP A1. Previous studies have shown that the ubiquitylation of hnRNP A1 at K29 was regulated by Elongin B/C-Cullin complexes 33, and the K63 ubiquitylation of hnRNP A1 at K3 and five lysine residues within RRM1 of hnRNP A1 was regulated by TRAF6 42.

Previous studies have demonstrated the functional roles of ZFP91 in promoting prostate, colon and pancreatic cancer cell proliferation, tumorigenesis and invasion 31, 32. In this study, we demonstrated the functions and mechanisms of ZFP91 in glucose metabolism programming in HCC cells. We found that ZFP91 suppresses cancer aerobic glycolysis in HCC cells by regulating the alternative splicing of glycolytic enzyme *PKM* pre-mRNA through hnRNP A1. Previous studies have shown that hnRNP A1 expression is regulated by c-myc at the transcriptional level and that the c-myc-hnRNP A1 pathway regulates PKM splicing to promote cancer aerobic glycolysis 11. In addition to the c-myc-induced transcription, we provide another fashion of the upregulation of hnRNP A1 protein levels in cancer cells in which the ubiquitin-mediated degradation of hnRNP A1 was impaired because the E3 ubiquitin ligase ZFP91 of hnRNP A1 was downregulated or absent in HCC cells.

In summary, we reveal a connection between ZFP91 and alternative splicing and cancer metabolism in HCC and find that ZFP91 is a potential tumor suppressor in HCC. ZFP91 suppresses cancer metabolism reprogramming and progression by ubiquitinating and degrading hnRNP A1 to regulate PKM splicing in HCC cells. ZFP91 serves as an independent prognostic biomarker for patients with HCC. Therapeutic targeting of the ZFP91-hnRNP A1 pathways may be an effective means of treating HCC.

## Materials and Methods

### Cell culture and tissue samples

HCC cell lines (BEL7402, SMMC7721, SK-hep1, MHCC-LM3 and MHCC97H) were obtained from the National Infrastructure of Cell Line Resources of China. The HEK-293T cell line was obtained from ATCC (CCL-2). SK-hep1, HCC-LM3, MHCC97H and HEK293T cells were cultured in DMEM medium supplemented with 100 units/mL penicillin, 100 units/mL streptomycin and 10% fetal bovine serum (FBS) at 37 °C in a 5% CO_2_ atmosphere, while BEL7402 and SMMC7721 were cultured in RPMI 1640 medium supplemented with 100 units/mL penicillin, 100 units/Ml streptomycin and 10% FBS at 37 °C in a 5% CO_2_ atmosphere. All these cells were periodically tested for mycoplasma by qPCR.

Six pairs of HCC tissue samples and their corresponding adjacent nontumor liver tissue samples were collected from the second affiliated Hospital of Guangzhou Medical University. This cohort was composed of 4 males and 2 females with median age of 49 years old. All selected patients with HCC had not been preoperatively treated by anticancer therapy.

Each patient with HCC who was involved in the investigation had obtained informed consent, and the collection of tissue samples had also been approved by the Internal Review and Ethics Committee of the Third Affiliated Hospital of Guangzhou Medical University. Tissue microarray chips containing HCC tissue samples and paired adjacent liver tissue samples and corresponding clinical pathology data were obtained from Shanghai OUTDO Biotech Co., Ltd. (Shanghai, China).

### Western blotting assays

Protein expression in cells was analyzed by standard Western blot assays. Western blotting was performed using anti-ZFP91 (PA5-41199, Thermo Fisher, 1:2000), anti-hnRNP A1 (11176-1-AP, Proteintech, 1:2000), anti-β-actin (BS6007M, Bioworld, 1:5000), anti-HA (561, MBL, 1:2000), anti-Flag (M185-3L, MBL, 1:2500), anti-PKM1 (#7067, CST, 1:1000), and anti-PKM2 (15822-1-AP, Proteintech, 1:1500). Band intensity was quantified using ImageJ software (NIH, Bethesda).

### Immunohistochemistry (IHC) staining assay

As previously described, IHC staining assays were performed on tissue microarray chips with anti-ZFP91 antibodies (HPA024037, SIGMA, 1:500) 43. All IHC samples were evaluated by two independent pathologists who were blinded to the sample origins and the subject outcomes. The expression of ZFP91 was evaluated using the semiquantitative German scoring system previously described 44, 45, and ZFP91 protein level (score) was assessed based on the staining intensity and the staining region 14. Scores <4 were indicative of low ZFP91 expression, and scores ≥4 were indicative of high ZFP91 expression.

### Migration and invasion assays

Transwell chambers (8 μm pore size, BD Biosciences) were used to perform cell migration and invasion assays *in vitro*, as previously described 46. In brief, HCC cells were transfected with the indicated siRNAs or plasmids. For migration assays, cells with serum-free medium were seeded into the upper chambers. The upper chambers were coated with Matrigel for invasion assays. Migrated and invasive cells were fixed with methanol and stained with 5% crystal violet. Images were captured from each membrane under a microscope, calculating the number of migratory.

### Cell growth assays

HCC cells (1×10^4^) were transfected with the indicated siRNAs or plasmids for 24 h and then plated in 24-well culture plates and cultured. Cells were counted at 24, 48, 72, 96 and 120 h (n = 3).

### Colony formation assays

Eight hundred HCC cells were transfected with the indicated plasmids or siRNAs for 24 h and then plated in 6-well culture plates and cultured in DMEM/1640 medium supplemented with 10% FBS for 14 days. These cells were fixed with methanol and then stained with crystal violet solution. Colonies containing ≥30 cells were counted under the microscope.

### Construction of cell lines with stable ZFP91 silencing

HCC SK-hep1 cells were infected with the lentivirus pLV5-GFP-Luc. SK-hep1-Luc cell line stably expressing luciferase was established. The lentivirus pLV3-ZFP91 shRNA that expresses ZFP91 shRNA was from GenePharma (Shanghai, China). The Luc-labeled SK-hep1-Luc cells were further transduced with lentivirus pLV3-ZFP91 shRNA. SK-hep1-Luc-shZFP91 cell line was constructed. ZFP91 knockdown was validated by western blotting.

### Animal experiments

Male BALb/c null and NOD-SCID mice (4-5 weeks old) were purchased from Charles River Laboratories in China (Beijing) and raised and maintained under specified conditions in the Animal Experiment Center of the College of Medicine (SPF grade), Jinan University. Mice were randomly assigned to the experimental group by researchers who were blinded to the subject outcomes. For the *in vivo* tumor growth assay, 1×10^6^ SK-hep1-Luc-NC or SK-hep1-Luc-ZFP91 shRNA-transduced HCC cells were subcutaneously injected into the left and right dorsal flanks of each mouse, respectively (n = 6). A month later, the tumors were dissected and weighed after the mice were euthanized. The *in vivo* lung metastasis assays were performed as previously described 14, 44. SK-hep1-Luc-NC or SK-hep1-Luc-ZFP91 shRNA-transduced HCC cells (2 × 10^6^ cells in 200 μL PBS) were injected into NOD-SCID mice in each experimental group via their tail veins. Two months after tumor implantation, the metastatic foci were visualized.

All mouse experiments were approved by the Laboratory Animal Ethics Committee of the Third Affiliated Hospital of Guangzhou Medicine University and Jinan University and conformed to the legal mandates and national guidelines for the care and maintenance of laboratory animals.

### Coimmunoprecipitation (Co-IP)

HEK-293T or HCC cells were transfected with the indicated vectors for 48 h, and Co-IP was subsequently performed using anti-HA or Flag antibodies (MBL). Then, the immune complexes were captured by protein A/G agarose beads (Santa Cruz, USA) and separated by SDS-PAGE. The SDS-PAGE gels were subsequently stained with silver or the proteins in co-immunoprecipitated complexes were detected by Western blotting.

### Protein identification by mass spectrometry

The differential gel bands and their corresponding negative gel bands were excised for in-gel trypsin digestion. Subsequently, the extracted peptides were analyzed using nano-LC-MS/MS (AB SCIEX TripleT OF 5600, USA), as previously described 14, 45.

### *In vivo* ubiquitination assay

Cells were cotransfected with the indicated plasmids for 36 h. Cells were then treated with MG132 (10 μM) for 8 h. The hnRNPA1-Flag or hnRNPA1-Flag K8R protein was immunoprecipitated using anti-Flag antibody, and the ubiquitination levels of hnRNPA1-Flag or hnRNPA1-Flag K8R protein were detected by Western blotting.

### *PKM* splicing assays

*PKM* splicing was analyzed as previously mentioned 14. In brief, total RNA was extracted by TRIzol. RNA reverse transcription was performed using the PrimeScript RT reagent Kit with gDNAEraser (TAKARA). cDNA obtained from reverse transcription was amplified by PCR and digested using PstI. Finally, the digested mixtures were separated by 8% nondenaturing PAGE.

### Measurement of lactate production

HCC cells were transfected with the indicated siRNAs or plasmids for 36 h and then incubated in phenol red-free DMEM medium without FBS for 4 h. The accumulation of lactate in the media was detected using a Lactate Colorimetric Assay kit II (K627-100, BioVision, USA). The background was corrected by deducting the OD value from the fresh phenol red-free medium. According to the lactate standard measurement values, the standard curve of nmol/well on OD_450 nm_ was drawn. The sample OD_450 nm_ values were applied to the standard curve to calculate the concentrations of lactate in test samples (n = 3).

### Glucose uptake assay

HCC cells were transfected with the indicated siRNAs or plasmids for 36 h and incubated in DMEM medium without L-glucose and phenol red for 8 h. The amount of glucose in these media was measured using a Glucose Colorimetric Assay kit (K606-100, Bio Vision, USA). Fresh DMEM medium was used for the negative control. Three biological replicates were performed.

### Statistical analysis

Statistical analyses were performed using SPSS program or Prism 5 software. T-test or U-test was used to analyze the experimental results between groups, and Pearson's chi-square test was used to analyze the correlations between ZFP91 levels and clinicopathological parameters. Two-way ANOVA was used to analyze the growth curve comparison. The Kaplan-Meier method was used to analyze overall survival (OS), and the independent predictors associated with OS were analyzed using the Enter (conditional) Cox proportional hazard model. All statistical data were expressed as the mean ± SD (mean ± SD) unless otherwise specified, where *P < 0.05, **P < 0.01 or ***P < 0.001 was considered to be statistically significant.

## Supplementary Material

Supplementary figures, table, data.

## Figures and Tables

**Table 1:** ZFP91 is an independent prognosis factor for OS for patients with HCC

Clinical character	OS (months)			
	Univariate analysis		Multivariatie analysis	
	HR (95% CI)	*P* value*	HR (95% CI)	*P* value^*^
Age (>50y vs. ≤ 50)	1.092 (0.620-1.924)	0.761	1.032 (0.548-1.945)	0.921
Gender (Female vs. Male)	1.715 (0.770-3.820)	0.187	2.523 (0.94606.730)	0.064
Cirrhosis (yes vs. no)	0.823 (0.369-1.837)	0.635	0.369 (0.129-1.055)	0.063
Histological Grade (G1-2,G2 vs. G2-3,G3)	0.957 (0.526-1.740)	0.886	0.617 (0.318-1.196)	0.153
TNM stage (I vs. II)	1.627 (0.926-2.858)	0.09	0.986 (0.325-2.987)	0.980
Tumor size (≤ 3 cm vs. > 3 cm)	2.273 (1.159-4.457)	0.017	3.690 (1.601-8.503)	0.002
Tumor number (≤ 3 n vs. > 3 n)	1.504 (0.767-2.947)	0.235	0.971 (0.471-2.005)	0.938
AFP (<400 μg/L vs. 400 μg/L)	1.790 (1.017-3.151)	0.044	0.569 (0.277-1.168)	0.124
Cirrhosis Nodule size (≤ 3 mm vs. > 3mm)	1.093 (0.622-1.920)	0.757	0.691 (0.356-1.341)	0.274
Clinical stage (I vs. II)	1.888 (1.069-3.337)	0.029	1.006 (0.336-3.016)	0.992
Recurrence (yes vs. no)	6.461 (2.884-14.473)	0.000	9.474 (3.680-24.392)	0.000
ZFP91	0.256 (0.127-0.515)	0.000	0.320 (0.149-0.689)	0.004

*Cox multivariate proportional hazard model.

## References

[1] Torre LA, Freddie B, Siegel RL, Jacques F, Joannie LT, Ahmedin J (2015). Global cancer statistics, 2012. Ca A Cancer Journal for Clinicians.

[2] Poon RTP (2011). Prevention of recurrence after resection of hepatocellular carcinoma: a daunting challenge. Hepatology.

[3] Tralhão JG, Tralhão JG, Dagher I, Lino T, Roudié J, Franco D (2007). Treatment of tumour recurrence after resection of hepatocellular carcinoma. Analysis of 97 consecutive patients. European Journal of Surgical Oncology.

[4] Zhao H, Yan G, Zheng L, Zhou Y, Sheng H, Wu L (2020). STIM1 is a metabolic checkpoint regulating the invasion and metastasis of hepatocellular carcinoma. Theranostics.

[5] Cui HZ, Dai GH, Shi Y, Chen L (2013). Sorafenib combined with TACE in advanced primary hepatocellular carcinoma. Hepatogastroenterology.

[6] Benjamin D, Cravatt B, Nomura D (2012). Global Profiling Strategies for Mapping Dysregulated Metabolic Pathways in Cancer. Cell Metabolism.

[7] Ravindran Menon D, Hammerlindl H, Torrano J, Schaider H, Fujita M (2020). Epigenetics and metabolism at the crossroads of stress-induced plasticity, stemness and therapeutic resistance in cancer. Theranostics.

[8] Bensaad K, Harris AL (2014). Hypoxia and Metabolism in Cancer. Advances in Experimental Medicine & Biology.

[9] Ken G (2006). Energy deregulation: licensing tumors to grow. Science.

[10] Lunt SY, Heiden MGV (2011). Aerobic Glycolysis: Meeting the Metabolic Requirements of Cell Proliferation. Annu Rev Cell Dev Biol.

[11] David CJ, Mo C, Marcela A, Peter C, Manley JL (2010). HnRNP proteins controlled by c-Myc deregulate pyruvate kinase mRNA splicing in cancer. Nature.

[12] Liu MX, Jin L, Sun SJ, Liu P, Feng X, Cheng ZL (2018). Metabolic reprogramming by PCK1 promotes TCA cataplerosis, oxidative stress and apoptosis in liver cancer cells and suppresses hepatocellular carcinoma. Oncogene.

[13] Israelsen WJ, Heiden MGV (2015). Pyruvate kinase: function, regulation and role in cancer. Seminars in Cell & Developmental Biology.

[14] Huang JZ, Chen M, Chen D, Gao XC, Zhu S, Huang H (2017). A Peptide Encoded by a Putative lncRNA HOXB-AS3 Suppresses Colon Cancer Growth. Molecular Cell.

[15] Yang W, Lu Z (2013). Regulation and function of pyruvate kinase M2 in cancer. Cancer Letters.

[16] Chen M, Sheng XJ, Qin YY, Zhu S, Wu QX, Jia L (2019). TBC1D8 Amplification Drives Tumorigenesis through Metabolism Reprogramming in Ovarian Cancer. Theranostics.

[17] Clower CV, Deblina C, Zhenxun W, Cantley LC, Heiden MGV, Krainer AR (2010). The alternative splicing repressors hnRNP A1/A2 and PTB influence pyruvate kinase isoform expression and cell metabolism. Proceedings of the National Academy of Sciences of the United States of America.

[18] Chen M, David CJ, Manley JL (2012). Concentration-dependent control of pyruvate kinase M mutually exclusive splicing by hnRNP proteins. Nat Struct Mol Biol.

[19] Chen M, Zhang J, Manley JL (2010). Turning on a fuel switch of cancer: hnRNP proteins regulate alternative splicing of pyruvate kinase mRNA. Cancer Res.

[20] Wang H (2013). Targeting the ubiquitin-proteasome system for cancer therapy. Cancer Science.

[21] Aaron C (2005). Early work on the ubiquitin proteasome system, an interview with Aaron Ciechanover. Interview by CDD. Cell Death & Differentiation.

[22] Xu SH, Zhu S, Wang Y, Huang JZ, Chen M, Wu QX (2018). ECD promotes gastric cancer metastasis by blocking E3 ligase ZFP91-mediated hnRNP F ubiquitination and degradation. Cell Death Dis.

[23] Li X, Zhong L, Wang Z, Chen H, Liao D, Zhang R (2018). Phosphorylation of IRS4 by CK1γ2 promotes its degradation by CHIP through the ubiquitin/lysosome pathway. Theranostics.

[24] Mai J, Zhong ZY, Guo GF, Chen XX, Xiang YQ, Li X (2019). Polo-Like Kinase 1 phosphorylates and stabilizes KLF4 to promote tumorigenesis in nasopharyngeal carcinoma. Theranostics.

[25] Johnson DE (2015). The ubiquitin-proteasome system: opportunities for therapeutic intervention in solid tumors. Endocrine-related cancer.

[26] Li W, Bengtson MH, Ulbrich A, Matsuda A, Reddy VA, Orth A (2008). Genome-Wide and Functional Annotation of Human E3 Ubiquitin Ligases Identifies MULAN, a Mitochondrial E3 that Regulates the Organelle's Dynamics and Signaling. Plos One.

[27] Anupama P, Young MA, Donato NJ (2014). Emerging potential of therapeutic targeting of ubiquitin-specific proteases in the treatment of cancer. Cancer Research.

[28] Liao Y, Liu Y, Xia X, Shao Z, Huang C, He J (2020). Targeting GRP78-dependent AR-V7 protein degradation overcomes castration-resistance in prostate cancer therapy. Theranostics.

[29] Jin X, Jin HR, Jung HS, Lee SJ, Lee JH, Lee JJ (2010). An atypical E3 ligase zinc finger protein 91 stabilizes and activates NF-kappaB-inducing kinase via Lys63-linked ubiquitination. Journal of Biological Chemistry.

[30] Unoki M, Okutsu JY (2003). Identification of a novel human gene, ZFP91, involved in acute myelogenous leukemia. International Journal of Oncology.

[31] Paschke L, Jopek K, Szyszka M, Tyczewska M, Ziolkowska A, Rucinski M (2016). ZFP91: A Noncanonical NF-κB Signaling Pathway Regulator with Oncogenic Properties Is Overexpressed in Prostate Cancer. Biomed Res Int.

[32] Ma J, Mi C, Wang KS, Lee JJ, Jin X (2016). Zinc finger protein 91 (ZFP91) activates HIF-1αviaNF-κB/p65 to promote proliferation and tumorigenesis of colon cancer. Oncotarget.

[33] Wang F, Fu X, Chen P, Wu P, Fan X, Li N (2017). SPSB1-mediated HnRNP A1 ubiquitylation regulates alternative splicing and cell migration in EGF signaling. Cell Research.

[34] Yang H, Zhu R, Zhao X, Liu L, Zhou Z, Zhao L (2019). Sirtuin-mediated deacetylation of hnRNP A1 suppresses glycolysis and growth in hepatocellular carcinoma. Oncogene.

[35] Xu SH, Zhu S, Wang Y, Huang JZ, Chen M, Wu QX (2018). ECD promotes gastric cancer metastasis by blocking E3 ligase ZFP91-mediated hnRNP F ubiquitination and degradation. Cell Death & Disease.

[36] Christofk HR, Vander MH, Wu N, Asara JM, Cantley LC (2008). Pyruvate kinase M2 is a phosphotyrosine-binding protein. Nature.

[37] Patel S, Woodgett J (2008). Glycogen synthase kinase-3 and cancer: good cop, bad cop?. Cancer Cell.

[38] Wang XQ, Ongkeko WM, Chen L, Yang ZF, Lu P, Chen KK (2010). Octamer 4 (Oct4) mediates chemotherapeutic drug resistance in liver cancer cells through a potential Oct4-AKT-ATP-binding cassette G2 pathway. Hepatology.

[39] Kotliarova S, Pastorino S, Kovell LC, Kotliarov Y, Song H, Zhang W (2008). Glycogen synthase kinase-3 inhibition induces glioma cell death through c-MYC, nuclear factor-kappaB, and glucose regulation. Cancer Res.

[40] Tang QL, Xie XB, Wang J, Chen Q, Han AJ, Zou CY (2012). Glycogen synthase kinase-3beta, NF-kappaB signaling, and tumorigenesis of human osteosarcoma. J Natl Cancer Inst.

[41] Mishra R (2010). Glycogen synthase kinase 3 beta: can it be a target for oral cancer. Mol Cancer.

[42] Fang J, Bolanos LC, Choi K, Liu X, Christie S, Akunuru S (2017). Ubiquitination of hnRNPA1 by TRAF6 links chronic innate immune signaling with myelodysplasia. Nat Immunol.

[43] Xiao CL, Zhu S, He M, Chen D, Zhang Q, Chen Y (2018). N(6)-Methyladenine DNA Modification in the Human Genome. Mol Cell.

[44] Zhu S, Wang JZ, Chen D, He YT, Meng N, Chen M (2020). An oncopeptide regulates m(6)A recognition by the m(6)A reader IGF2BP1 and tumorigenesis. Nat Commun.

[45] Meng N, Chen M, Chen D, Chen XH, Wang JZ, Zhu S (2020). Small Protein Hidden in lncRNA LOC90024 Promotes "Cancerous" RNA Splicing and Tumorigenesis. Adv Sci (Weinh).

[46] Huang JZ, Chen M, Zeng M, Xu SH, Zou FY, Chen D (2016). Down-regulation of TRPS1 stimulates epithelial-mesenchymal transition and metastasis through repression of FOXA1. J Pathol.

