# Supplementary material for: E3 ligase ZFP91 inhibits Hepatocellular Carcinoma Metabolism Reprogramming by regulating PKM splicing
Source: Theranostics. 2020 Jul 9;10(19):8558–72. doi: 10.7150/thno.44873 (PMC7392027; doi:10.7150/thno.44873)
Supplement: Supplementary file 1 — Supplementary figures, table, data. [file thnov10p8558s1.pdf]

Supplementary Information

Supplementary Figures

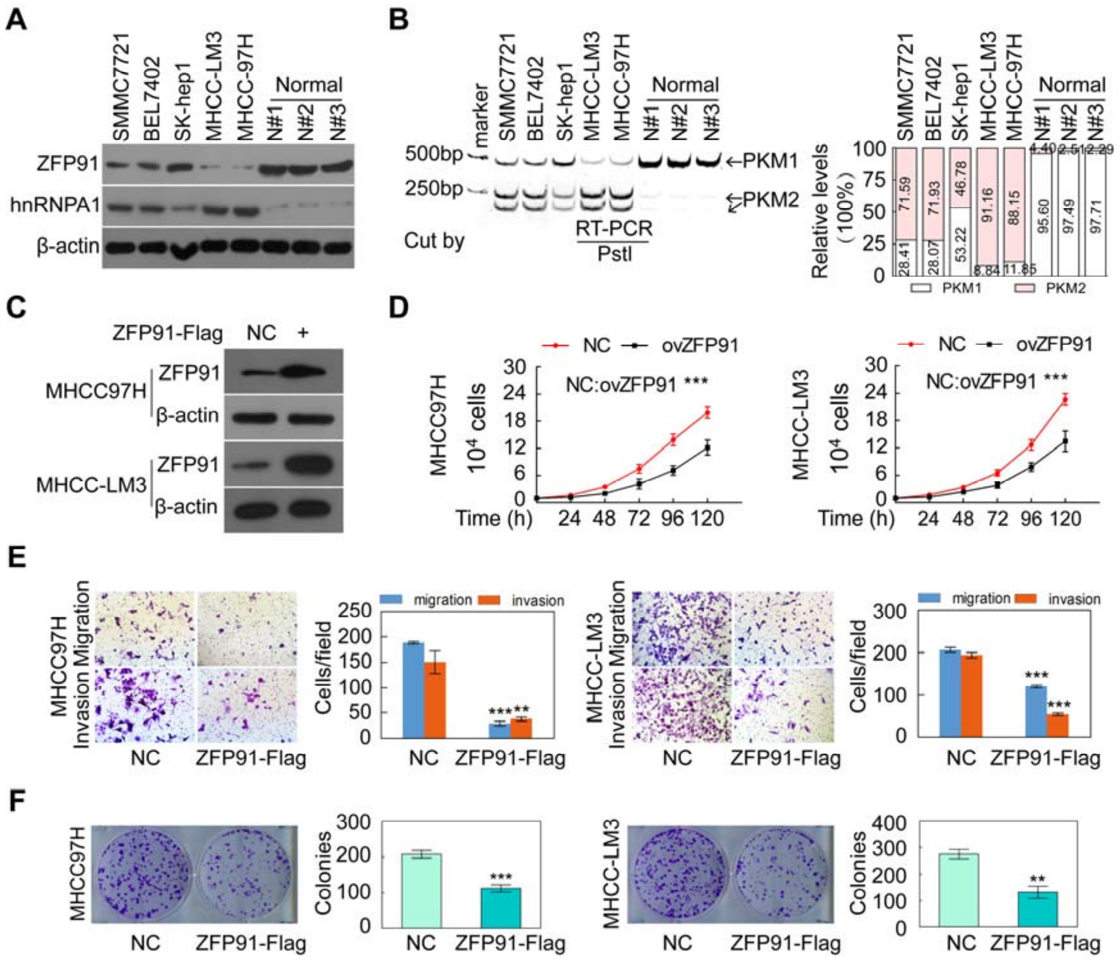

Figure S1. ZFP91 overexpression suppresses HCC cell growth, colony formation, migration and invasion. (A) The ZFP91 and hnRNP A1 protein levels and PKM pre-mRNA splicing in both five HCC cell lines and three normal liver tissues were detected. (B-E) MHCC97H and MHCC-LM3 cells were transfected with ZFP91-Flag plasmids; ZFP91 protein level (B), cell growth (C), migration and invasion (D), and colony formation (E) was determined. Data are represented as mean ± SD.

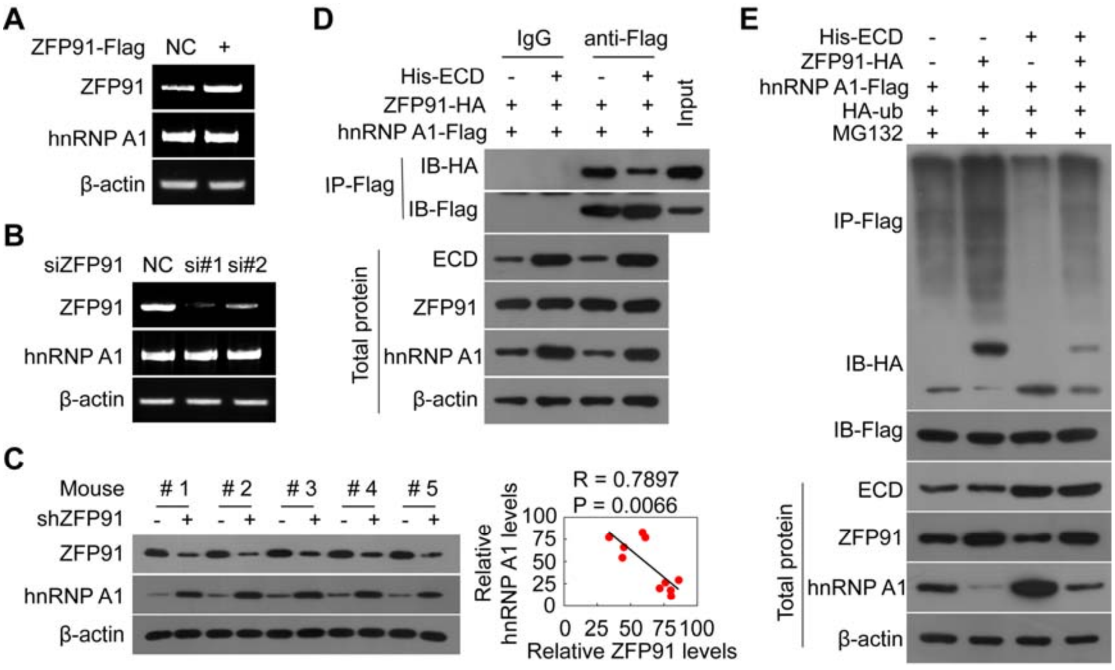

Figure S2. ZFP91 decreased hnRNP A1 protein level, not mRNA level, and ECD attenuated the interaction of ZFP91 with hnRNP A1 and the ubiquitination of ZFP91 on hnRNP A1. (A) MHCC-LM3 HCC cells were transfected with ZFP91-Flag plasmid, and the hnRNP A1 mRNA level was determined. (B) SK-hep1 HCC cells were transfected with anti-ZFP91 siRNA, and the hnRNP A1 mRNA level was determined. (C) ZFP91 and hnRNP A1 protein levels in mouse xenograft tumor tissues in Figure 2E were detected. The correlations of ZFP91 with hnRNP A1 were analyzed in the right panel. (D) MHCC-LM3 HCC cells were transfected with the indicated plasmids, hnRNP A1-Flag complexes were co-immunoprecipitated with anti-Flag, and ZFP91-HA was detected using anti-HA antibody. (E) MHCC-LM3 HCC cells were transfected with the indicated plasmids for 36 h, followed by treatment with 10 μM MG132; the polyubiquitination level of hnRNP A1 was detected.

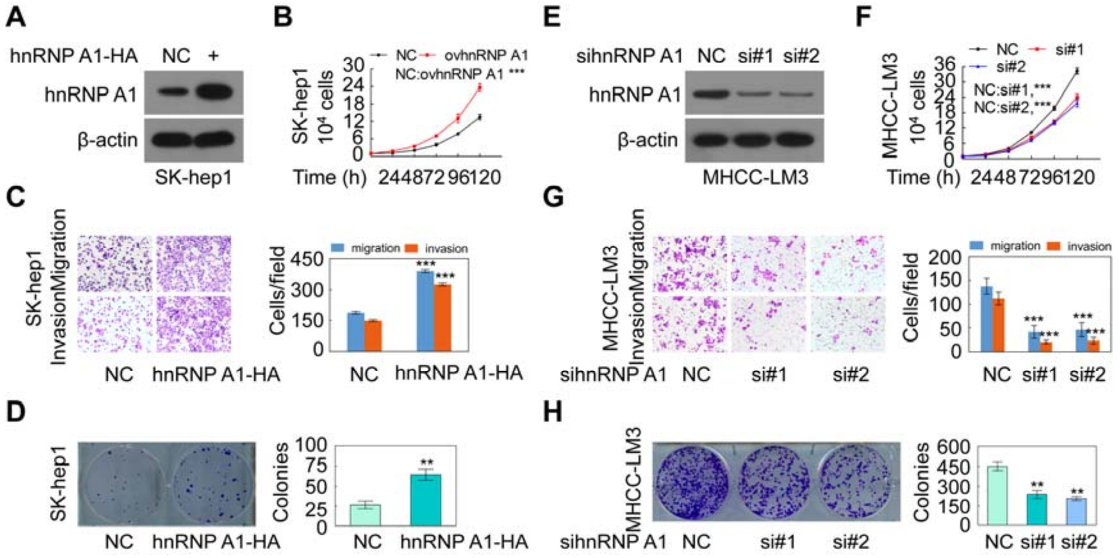

Figure S3. The ubiquitination modification lysine 8 site in the hnRNP A1 protein was identified by mass spectrometry. (A) Two representative MS/MS spectra of the identification the lysine 8 (K8) ubiquitination site in the hnRNP A1 protein. (B) Matching of the MS-identified peptides and their post-modifications with the acid amino sequence of hnRNP A1 protein.

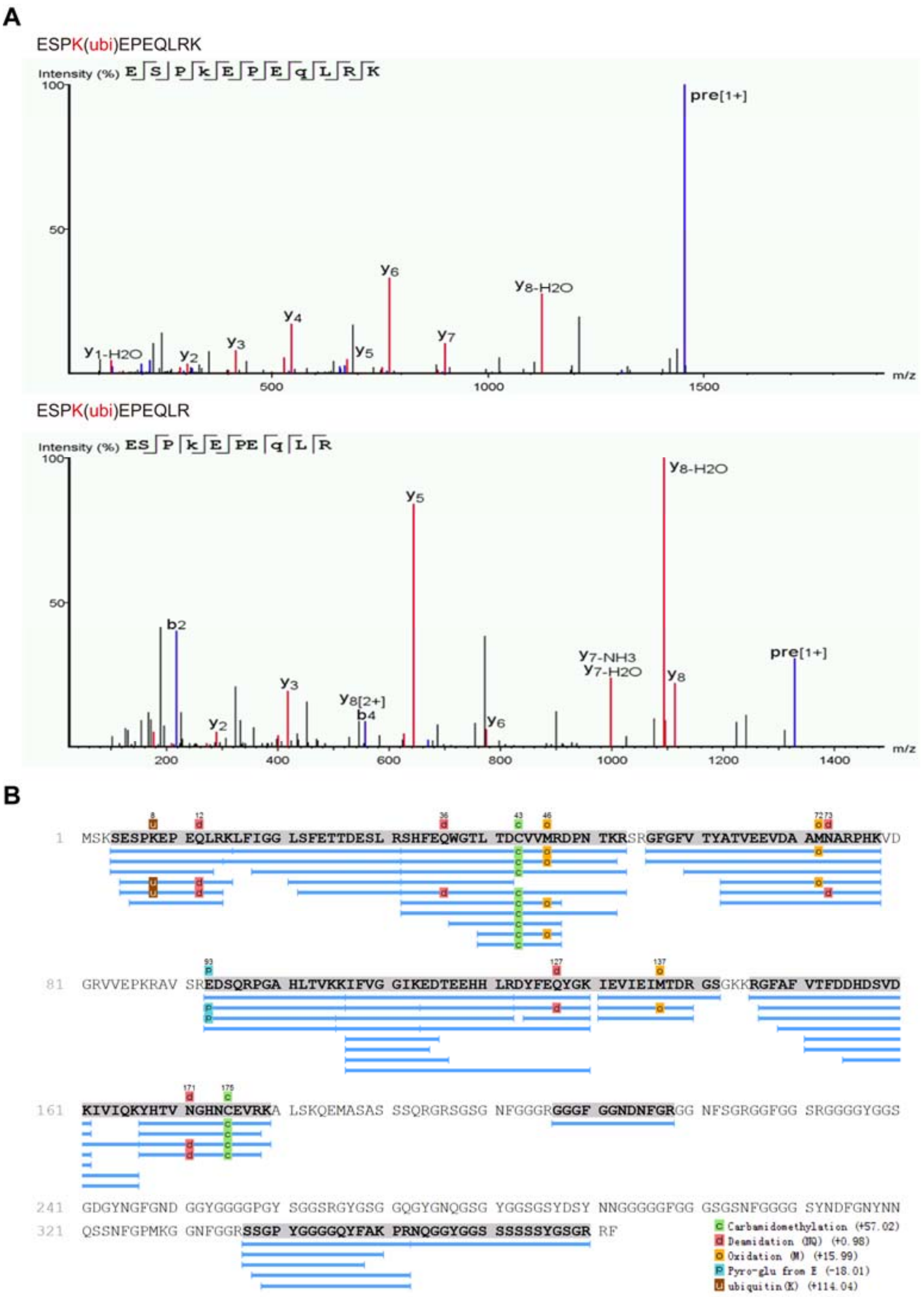

Figure S4. HnRNP A1 promotes HCC cell growth, colony formation, migration and invasion. (A-D) SK-hep1 cells were transfected with hnRNP A1-HA plasmids; hnRNP A1 protein level (A), cell growth (B), migration and invasion (C), and colony formation (D) were determined. (E-H) MHCC-LM3 cells were transfected with anti-hnRNP A1 siRNAs; hnRNP A1 protein level (E), cell growth (F), migration and invasion (G), and colony formation (H) were determined (n = 3). Data are represented as mean ± SD.

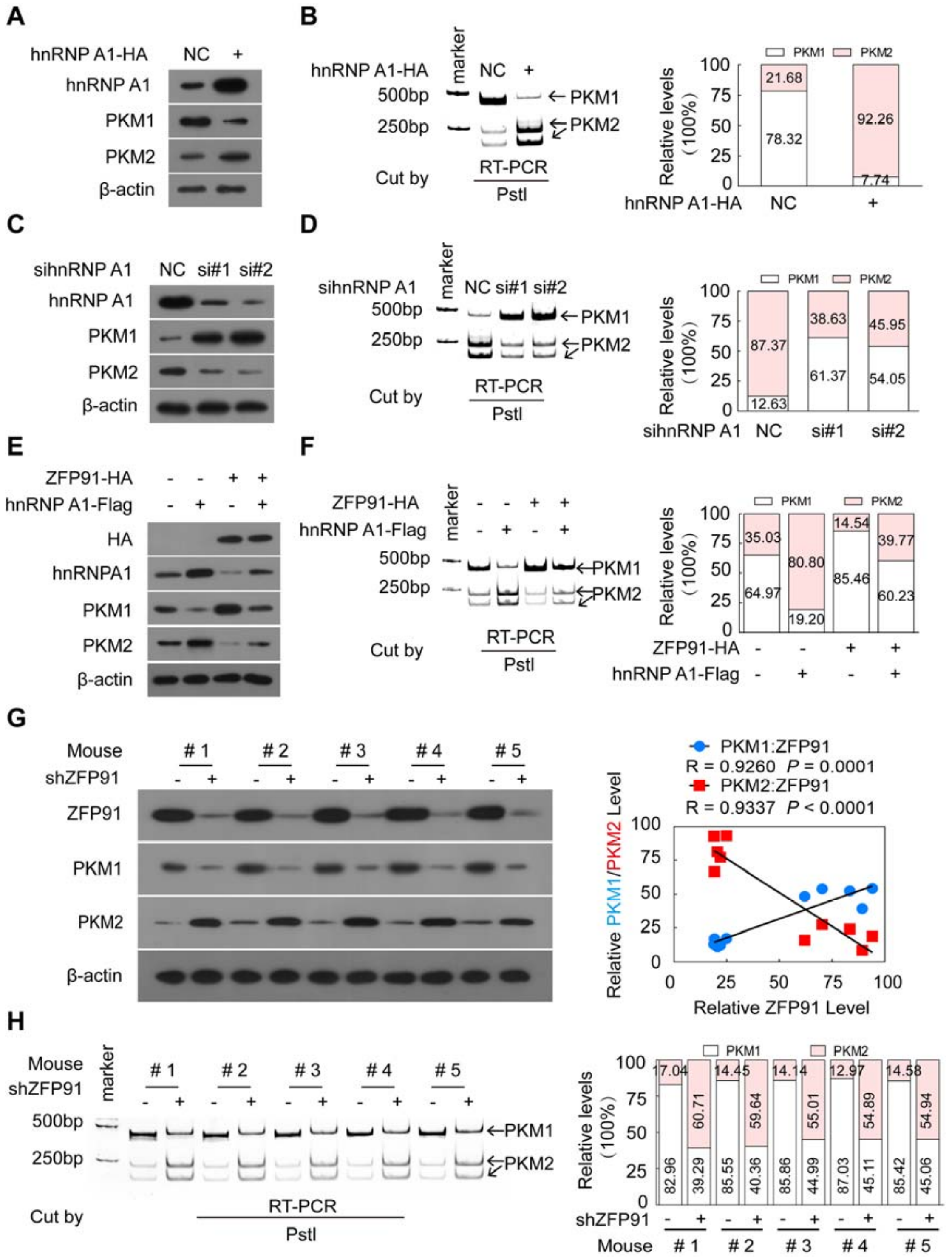

Figure S5. HnRNP A1 promotes the alternative splicing of PKM pre-mRNA through hnRNP A1. (A, B) HCC SK-hep1 cells were transfected with hnRNP A1-HA plasmids; the protein levels of PKM1 and PKM2 (A) and PKM pre-mRNA splicing (B) were detected. (C, D) HCC MHCC-LM3 cells were transfected with anti-hnRNP A1 siRNAs; the protein levels of PKM1 and PKM2 (C) and PKM pre-mRNA splicing (D)

were detected. (E, F) Both ZFP91-HA and hnRNP A1-Flag plasmids were cotransfected into MHCC-LM3 cells; the protein levels of PKM1 and PKM2 (E) and PKM pre-mRNA splicing (F) were detected. (G, H) The protein levels of PKM1 and PKM2 (G) and PKM pre-mRNA splicing (H) were detected in mouse xenograft tumor tissues in Figure 2E. The correlations of ZFP91 protein levels with PKM1 and PKM2 protein levels were analyzed (right panel in G).

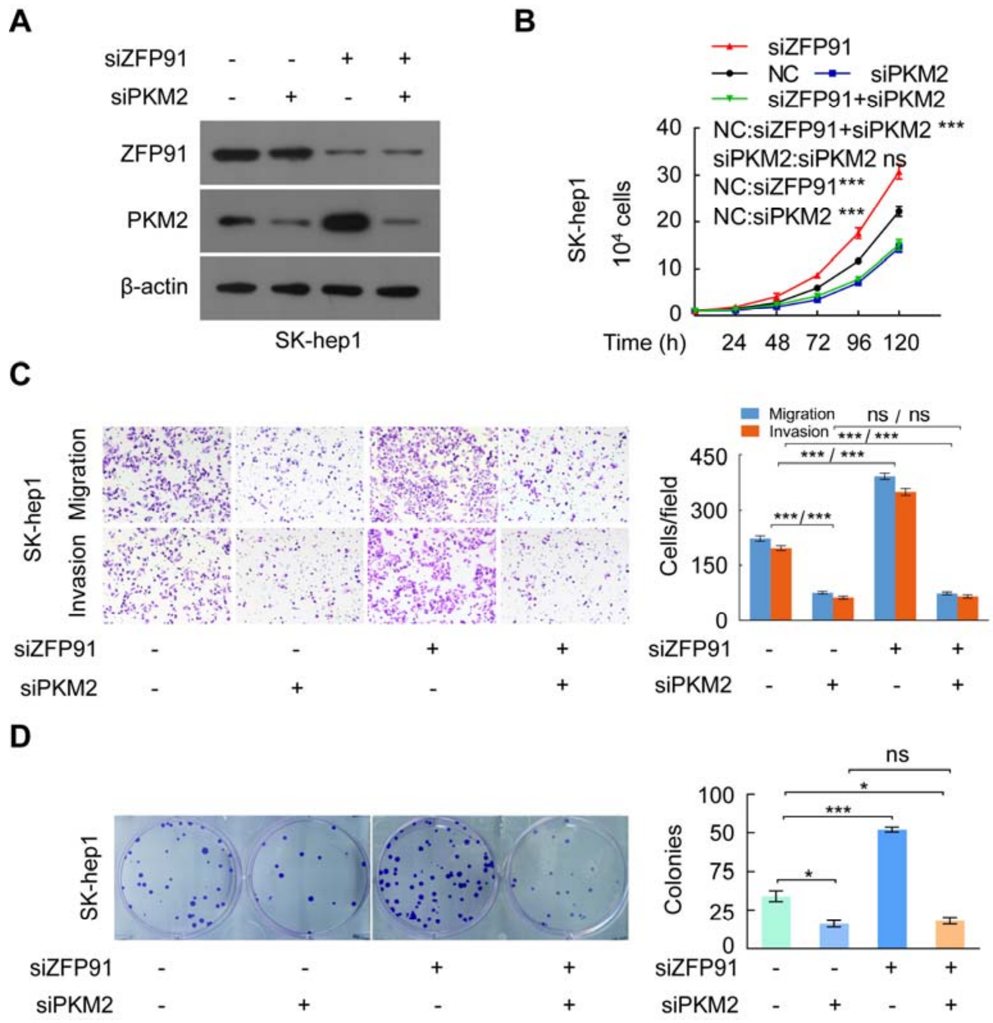

Figure S6. ZFP91 inhibits HCC cell proliferation, colony froamtion, migration and invasion through PKM2. HCC SK-hep1 cells were cotransfected with anti-ZFP91 siRNA together with anti-PKM2 siRNA; ZFP91 and PKM2 protein level (A), cell growth (B), migration and invasion (C), and colony formation (D) were determined (n = 3). Data are represented as mean ± SD.

Supplementary Tables

Table S1: Correlations between ZFP91 protein levels and clinicopathological features in 90 HCC cases.

Clinical character	Clinical groups	All cases	ZFP91		x^2	*P* value*
			Low	High		
Age (Years)	≤ 50	42	29	13	2.686	0.101
	＞ 50	48	25	23		
Gender	Female	16	8	8	0.811	0.368
	Male	74	46	28		
Cirrhosis	YES	78	47	31	0.016	0.899
	NO	12	7	5		
Histological Grade	G1-2, G2	58	33	25	0.655	0.418
	G2-3, G3	32	21	11		
Tumor size (cm)	≤ 3	29	17	12	0.034	0.854
	＞ 3	61	37	24		
Tumor number (n)	≤ 3	74	43	31	0.621	0.431
	＞ 3	16	11	5		
AFP (μg/L)	＜ 400	57	32	25	0.965	0.326
	≥ 400	33	22	11		
Cirrhosis Nodule size (mm)	≤ 3	50	29	21	0.188	0.665
	＞ 3	40	25	15		
Clinical stage#	I	61	31	30	6.648	0.010
	II	29	23	6		
TNM stage	I	54	27	27	5.625	0.018
	II	36	27	9		
Recurrence of cancer	YES	37	17	20	5.171	0.023
	NO	53	37	16		

ZFP91 low score: 0-3; ZFP91 high score: 4-7;

*Pearson Chi-square test;

#Clinical stage: American Joint Committee on Cancer classifcation (AJCC).

siRNA sequences

ZFP91 si#1:

Sense: 5’- CAUUGCUGCAUCUAGACCUTT-3’

Anti-sense: 5’- AGGUCUAGAUGCAGCAAUGTT-3’

ZFP91 si#2:

Sense: 5’- GCAGCUCAUUUGCAAGUCATT-3’

Anti-sense: 5’- UGACUUGCAAAUGAGCUGCTT-3’

hnRNP A1 si#1:

Sense: 5’-GCUGUGUAAAGUUAGUCUATT-3’

Anti-sense: 5’-UAGACUAACUUUACACAGCTT-3’

hnRNP A2 si#2:

Sense: 5’-GUGUAGUUGAACUGAUAGUTT-3’

Anti-sense: 5’-ACUAUCAGUUCAACUACACTT-3’

NC siRNA:

Sense: 5’-GCACAAGCUGGAGUACAACUACATT -3’

Anti-sense: 5’- UGUAGUUGUACUCCAGCUUGUGCTT-3’

RT-PCR primers

ZFP91

Forward: TCCTTGCCCATCCTCGCTATT

Reverse: TGTTTGGCATGTCGCAGAAGT

β-actin

Forward: GAGAAAATCTGGCACCACACC

Reverse: GGATAGCACAGCCTGGATAGCAA

PKM

Forward: CTGAAGGCAGTGATGTGGCC

Reverse: ACCCGGAGGTCCACGTCCTC

Vector construction primers

ZFP91-Flag

Forward: CGGGGTACCGACGGACAAGCCCCGATGC

Reverse:

CGGAATTCTTACTTGTCATCGTCGTCCTTGTAGTCAGGTCCGGCAGAGTCTG

AATC

ZFP91-HA

Forward: CGGGGTACCGACGGACAAGCCCCGATGC

Reverse:

CGGAATTCTTAAGCGTAATCTGGAACATCGTATGGGTAAGGTCCGGCAGA

GTCTGAATC

hnRNP A1-Flag

Forward: CGGGGTACCACGTTCGTCAGCTTGCTCCTT

Reverse:

CGGAATTCTTACTTGTCATCGTCGTCCTTGTAGTCAAATCTTCTGCCACTGC

CATAGC

hnRNP A1-HA

Forward: CGGGGTACCACGTTCGTCAGCTTGCTCCTT

Reverse:

CGGAATTCTTAAGCGTAATCTGGAACATCGTATGGGTAAAATCTTCTGCCAC

TGCCATAGC

hnRNP A1-Flag K8R mutation

Forward: TGCCGTCAGATCTAAGTCAGAGTCTCCTAAAGAGCCC

Reverse: ACTTAGATCTGACGGCAGGGTGAAGAGAGACT
